# α-Tocopherol Improves Microcirculatory Dysfunction on Fructose Fed Hamsters

**DOI:** 10.1371/journal.pone.0134740

**Published:** 2015-08-05

**Authors:** Beatriz C. S. Boa, Carlos M. M. R. Barros, Maria das Graças C. Souza, Raquel C. Castiglione, Fátima Z. G. A. Cyrino, Eliete Bouskela

**Affiliations:** Laboratory for Clinical and Experimental Research on Vascular Biology (BioVasc), Biomedical Center, State University of Rio de Janeiro, Rio de Janeiro, Brazil; Universidade Federal do Rio de Janeiro (UFRJ), BRAZIL

## Abstract

Fructose, an everyday component of western diet associated to chronic hyperglycemia and enhanced free radical production, impairs endothelial function and supplementation with antioxidants might improve it. In this study we investigated if vitamin E could reverse the microvascular damage elicited by fructose. Male Syrian golden hamsters drank either 10% fructose solution (F) or filtered water (C), combined with three concentrations of vitamin E in their chows [zero, normal (VE) or 5X (5XVE)] during 60 days. Microvascular reactivity in response to topical application of acetylcholine (Ach; endothelium-dependent vasodilator) or sodium nitroprusside (SNP; endothelium-independent vasodilator) and macromolecular permeability increase induced by either 30 min ischemia followed by reperfusion (I/R) or topical application of histamine (5 μM) were assessed using the cheek pouch preparation. Compared to controls (drinking filtered water), fructose-drinking animals showed decreased vasodilatation to acetylcholine in all concentrations tested (-56.2% for 10^-9^M, -53.9% for 10^-7^M and -43.7% for 10^-5^M). On the other hand, vitamin E supplementation resulted in increased responses for both water and fructose drinking groups (177.4% for F vs. F/5XVE and 241.6% for C vs. C/5XVE for 10^-5^M Ach). Endothelial-independent vasodilatation explored by topical application of SNP was restored and even enhanced with the supplementation of 5X vitamin E in both groups (80.1% for F vs. F/5XVE; 144.2% for C vs. C/5XVE; 3.4% of difference for C/5XVE vs. F/5XVE on 10^-5^M SNP). The number of leaky sites after I/R and histamine stimuli in vitamin E supplemented animals decreased (-25.1% and -15.3% for F vs. F/5XVE; and -21.7% and -16% of leaky sites comparing C vs. C/5XVE, respectively for I/R and histamine stimuli) pointing to tightening of the endothelial barrier for macromolecular permeability. Our results strongly suggest that vitamin E could improve the endothelial function and permeability barrier and also reverse impairments elicited by sugar overload.

## Introduction

Humans present a tendency to choose more palatable diets and sugars like fructose and glucose function as daily sweeteners. Given the substantial participation of fructose in Western diet, it seems important to elucidate its metabolic effects, as well as its potentials cardiovascular risks. Diabetes mellitus (DM) is a metabolic disorder characterized by chronic hyperglycemia, resulting from defects on either insulin secretion or action. The scenario of prolonged hyperglycemia is associated to permanent damage, dysfunction and failure of various tissues and organs, including kidneys, nerves and retina [1–3]. These clusters of abnormalities are associated to higher incidence of cardiovascular morbidity and mortality. Moreover it is known that oxidative stress, one outcome of chronic hyperglycemia, stands for an important contributor to cardiovascular failure in diabetic patients [4–6]. Although studies concerning the influence of diet pattern (i.e. fructose ingestion) on insulin action, glucose levels and its consequences on morbidity and mortality are extensive in the literature, few have demonstrated outcomes of therapeutic approaches directly on the microvasculature. The microcirculation could provide a primary evaluation spot, in which alterations in individual’s health might be localized, even in the absence of ill symptoms. The endothelium is essential for autoregulatory mechanisms and nitric oxide (NO) production plays an important role on vascular tone and health [7]. Endothelial dysfunction (ED) could be characterized by reduction in the bioavailability of vasodilators, mainly NO, and activation of endothelial cells elicited by predominant pro-inflammatory, proliferative and pro-coagulant milieu state [8].

Fructose ingestion can cause insulin resistance, hyperglycemia, and hypertriglyceridemia in rats [9]. These animals display different abnormalities, such as reduction in tritiated glucose uptake by adipocytes, reduction of endothelium-dependent vasodilatation induced by acetylcholine in aortic strips [10], reduction to 80% in tyrosine phosphorylation of IRS-1 in the soleus muscle [11], increase in fasting plasma insulin without hyperglycemia, decreased muscarinic receptors expression, increased dependence on nitric oxide (NO) and impairment of α_2_-adrenergic-mediated relaxation [12].

Diets containing mineral antioxidants such as zinc, selenium, copper and manganese could counteract the oxidative stress observed in diabetes mellitus through their catalytic activity on antioxidant enzymes [13]. Investigations using vitamin E as an antioxidant have shown its protective effect on vascular endothelium after ischemia followed by reperfusion [14], increase in antioxidant levels in brain cortex and liver, along with a decrease in fasting glucose, insulin, and insulin resistance in ovariectomized rats [15].

Based on the aforementioned information, the present investigation focused on evaluating the effects of different contents of vitamin E on microvascular damages and altered biochemical markers elicited by chronic substitution of the drinking water by 10% fructose solution. We have hypothesized that endothelial damages provoked by fructose overload may be either attenuated or reversed by antioxidant properties of vitamin E. If our hypothesis is correct, the use of this particular vitamin, as daily supplement, might function as future therapeutic approach to prevent microangiopathies.

## Material and Methods

### 3.1—Experimental methods

Eight weeks-old male golden hamsters (*Mesocricetus auratus*—approximate weight 100 g; n = 137) were used. All animals were obtained from colonies of Anilab (Paulínia, SP, Brazil) and housed in plastic cages under controlled light (12 h/ 12 h cycle) at constant temperature (21.5±0.5°C) with light from 06:00 to 18:00 h.

### 3.2—Experimental chow

The natural-ingredient diet containing different concentrations of vitamin E chows were used replacing standard feeding during the experimental period [zero, normal (75U/Kg) and 5X normal content of vitamin E (375U/kg)]. The used chow was developed by the Experimental Laboratory of Nutrition of Fluminense Federal University (UFF, Niterói, RJ, Brazil), made according to the American Institute of Nutrition Rodent Diets (AIN-93) recommendations [16].

**Fig 1:**
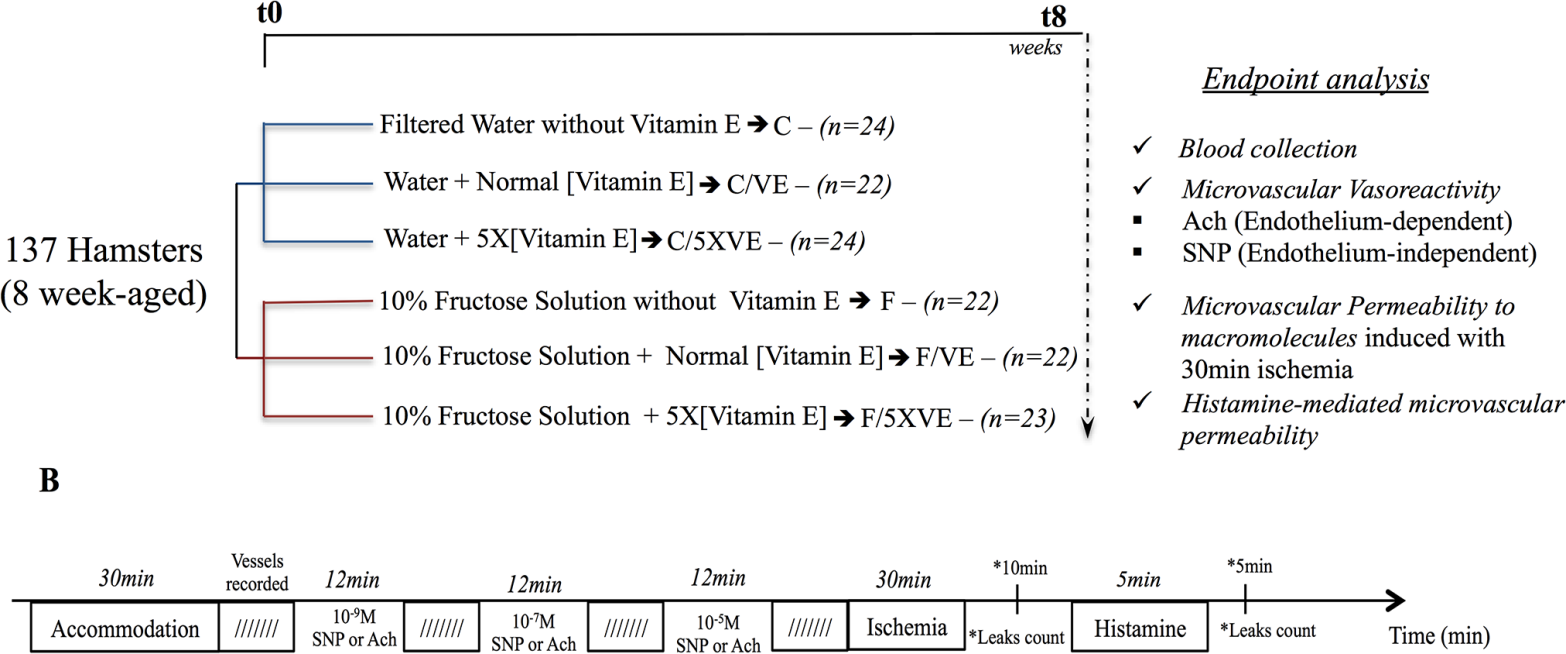
Experimental design and protocol. (1A) Eight weeks-old hamsters were treated during 8 weeks as described: animals were divided into two major groups, substitution of the drinking water by 10% fructose solution or kept drinking filtered water. Each major group had the formulated chow associated to three different concentrations of vitamin E: zero vitamin E (groups F and C), 75U/kg (normal concentration of vitamin E—groups F/VE and C/VE) and 375U/kg (5 times the normal concentration of vitamin E—groups F/5XVE and C/5XVE). After the 8^th^ week of treatment, the hamster cheek pouch microcirculation was evaluated by intravital microscopy and animals were euthanized for blood collection. (1B) Microcirculatory function was evaluated in two fronts: endothelial function by topical application of either acetylcholine or sodium nitroprusside, both in three different concentrations (10^−9^, 10^−7^ and 10^-5^M), in a cumulative dose-response curve and macromolecular permeability increase induced by either ischemia/reperfusion (30 min local ischemia followed by reperfusion) or topical application of histamine (5 μM during 5 min).

Nutritional characteristics of the experimental chow are exposed in Table 1. Water and rodent chow were autoclaved at the State University of Rio de Janeiro (Laboratory for Clinical and Experimental Research in Vascular Biology, Rio de Janeiro, Brazil) and provided *ad libitum*. Food and water intake were weekly monitored during the experimental period as exposed in Table 2. Experimental design and groups are presented on Fig 1.

**Table 1:** Contents of the diet used on treated hamsters.

Ingredient	g/kg diet
Cornstarch	397.486
Casein	200.000
Dextrinized cornstarch	132.000
Sucrose	100.000
Soybean oil	70.000
Fiber	50.000
Mineral Mix	35.000
Vitamin Mix*	10.000
L-Cystine	3.000
Choline	2.500
Tert-butylhydroquinone	0.014

*Vitamin Mix = Vitamin E content was altered in order to achieve three different concentrations [zero, (Normal) 75U/Kg and (5X times) 375U/Kg chow].

**Table 2:** Characteristics of investigated animals.

	C (n = 24)	F (n = 22)	C/VE (n = 22)	F/VE (n = 22)	C/5XVE (n = 24)	F/5XVE (n = 23)
	Mean ± SEM	Mean ± SEM	Mean ± SEM	Mean ± SEM	Mean ± SEM	Mean ± SEM
Body Weight (g)	112.6 ± 13	113.6 ± 26	115 ± 15.7	127.3 ± 36	118.6 ± 18	124.7 ± 34
Energy Intake (kJ/day/animal)	70.3 ± 9	51.8 ± 14	69.8 ± 7.3	56.7 ± 11	70.8 ± 9.8	58.9 ± 10
Fructose and Water ingestion (ml)	230 ± 41.8	281.3 ± 45	239.6 ± 37	275.9 ± 41	235 ±37.8	300.5 ± 44
Fasting blood Glucose (mg/dl)	71 ± 1.5	*143.3 ± 5.1	66.9 ± 1.2	+146.2 ± 1.6	76.9 ± 3.1	#152.6 ± 4.3
Fasting blood Insulin (UI/ml)	37.7 ± 5.7	*105.9 ± 16.9	45.3 ± 4.9	+114.5 ± 11.4	48.3 ±5.3	#132.4 ± 12.4
Cholesterol (mg/dl)	174 ± 2.9	168.4 ± 1.6	166.7 ± 3.1	165.8 ± 1.5	168.9 ± 2.5	161.3 ± 3.2
Blood triglycerides (mg/dl)	170 ± 3.4	172 ± 22	165.8 ± 12	160.2 ± 22	163.8 ± 13	159.9 ± 27
Heart rate (bpm)	303,7 ± 5.3	338.9 ± 7.8	299.7 ± 5.7	321.7 ± 2.4	311.4 ± 5.9	324.7 ± 7
Mean arterial pressure (mmHg)	117.5 ±5.3	126.9 ± 8.7	120.6 ± 5.4	128.1 ± 4.3	121.8 ± 4.8	132 ± 6.1

Measurements performed on the day of the experiment: weight, fasting blood glucose and insulin, triglycerides and cholesterol. All values are presented as mean ± standard error of the mean. C = Animals which drank filtered water and ingested chow without vitamin E during 8 weeks. C/VE = Animals which drank filtered water and ingested chow with 75U/kg of vitamin E during 8 weeks. C/5XVE = Animals which drank filtered water and ingested chow with 5 times the normal concentration of vitamin E (375U/kg) during 8 weeks. F = Animals which drank 10% fructose solution and ingested chow without vitamin E during 8 weeks. F/VE = Animals which drank 10% fructose solution and ingested chow with 75U/kg of vitamin E during 8 weeks. F/5XVE = Animals which drank 10% fructose solution and ingested chow with 5 times the normal concentration of vitamin E (375U/kg) during 8 weeks.

* Significantly different from C values (p<0.01).

+. Significantly different from C/VE values (p<0.01).

#Significantly different from C/5XVE values (p<0.01).

### 3.3—Ethic Statement

The protocol was approved by the Ethical Committee of the State University of Rio de Janeiro (CEUA/061/2010). The investigation was strictly conducted according to the *Guide for Care and Use of Laboratory Animals* published by the US National Institute of Health (NIH Publication No. 85–23, revised in 1996) as well as the guide of the Brazilian College of Animal Experimentation. Animals were euthanized through cardiac puncture, under anesthesia, used for blood analysis and all efforts were made to minimize their suffering.

All surgical procedures were performed under anesthesia induced by an intraperitoneal injection of 0.1–0.2 ml of sodium pentobarbital (Pentobarbital sodique, 60 mg/ml, Sanofi Santé Animale, Paris, France) and maintained with α-chloralose [100 mg/kg body weight (Sigma Chemicals, St. Louis MO, USA)] given intravenously through a cannula inserted into the femoral vein. A tracheal tube was inserted through tracheotomy to facilitate spontaneous breathing (room air). Throughout surgery and the whole experiment, hamsters were placed on a heating pad, controlled by a rectal thermistor, in order to maintain their body temperature at 37.5°C (LTB 750 Thermostat System, Uppsala, Sweden).

### 3.4—Microcirculatory research model

The hamster cheek pouch is an appropriate preparation to study effects of dietary changes provoked by sugar overload. The cheek pouch is an invagination of the oral mucosa under the subcutaneous tissue down to the shoulder region. Its blood supply comes mainly from the carotid arteries, although some blood is diverged to the retractor muscle, also part of the cheek pouch structure. The preparation remains stable for 5–6 h for arteriolar reactivity to acetylcholine (ACh) [17], for macromolecular permeability increase induced by bradykinin [18] and for spontaneous arteriolar vasomotion [19]. There are several advantages related to the use of this preparation: 1) ease and relatively non-traumatic access make it useful in studies requiring repeated observations of the same site; 2) highly vascularized with all classes of microcirculatory vessels visible within the microscopic field, allowing observation and comparison of various segments; 3) clarity and optical properties are good when compared to other densely vascularized tissues; 4) presence of skeletal muscle and cutaneous microcirculatory beds [17].

### 3.5—Intravital microscopy

The cheek pouch was gently mounted on an experimental chamber as previously described [19] where it was superfused at a rate of 4.0 ml/min (microvascular reactivity) and 6.0 ml/ml (ischemia/reperfusion and histamine) by a HEPES-supported HCO_3_^-^ saline solution [composition in mM: NaCl 110.0, KCl 4.7, CaCl_2_ 2.0, MgSO_4_ 1.2, NaHCO_3_ 18.0, N-2-hydroxyethylpiperazine-N’-2-ethanesulfonic acid (HEPES) 15.39 and HEPES Na^+^-salt 14.61] bubbled with 5% CO_2_-95% N_2_. With that, the pH of the solution was maintained at 7.4 and temperature was adjusted to 36.5°C, keeping physiological conditions for the experiments. The complete preparation was untouched for 30 min before measurements (resting period) placed under an intravital microscope (Leitz, Wetzlar, Germany, optical magnification x 210, NA 0.22) coupled to a closed-circuit TV system. Images were recorded in sVHS for future analysis.

### 3.6—Microvascular reactivity

To address changes on endothelium function three areas containing 2^nd^ to 3^rd^ order arterioles were chosen, taking into account their position (e.g. presence of fat cells and/or bifurcations), to return exactly to the same site for dose response evaluations after topical application of vasoactive drugs. As precaution, a conversion ruler (mm to μm) was used to guarantee that recorded vessels encompassed 2^nd^ to 3^rd^ order and mean luminal diameter of 60–80 μm was considered as upper limit. These measures helped to ensure that observed microvascular reactivity was similar in terms of endothelial responses. Mean internal microvessel diameters were determined using the Image Shearing device (Vista Electronics, model 908, San Diego, CA, USA), at baseline and after each topical application, 10 min each, of three concentrations of freshly prepared acetylcholine [Ach 10^−9^, Ach 10^−7^ and Ach 10^−5^ M (Sigma Chemicals, St. Louis, MO, USA)] or sodium nitroprusside [SNP 10^−9^, SNP 10^−7^ and SNP 10^−5^ M (Sigma Chemicals, St. Louis, MO, USA)] in a cumulative dose-response curve.

### 3.7—Microvascular permeability measurements

To check endothelial barrier integrity on the hamster cheek pouch, two processes were utilized to observe microvascular permeability to macromolecules: localized ischemia/reperfusion and histamine-induced leakage. The first one reflects the permeability increase provoked by reperfusion lesions, and could highlight how treatment with vitamin E could protect it from the over production of reactive oxygen species responsible for microvascular damages. The second one evaluates the possible protective feature of vitamin E on histamine-mediated microvascular permeability. Ischemia was initiated 30 min after the end of the microvascular reactivity protocol to allow stabilization of vessels after drug stimulation, using an air inflatable tourniquet distally placed around the neck of the pouch (HCP) inflated during 30 min to suppress local blood flow as previously described [18]. FITC-dextran (150 kDa, Bioflor HB, Uppsala, Sweden, i.v 25 mg/100 g body weight, 5% solution) was used for measurements of macromolecular permeability changes at post-capillary venules [20]. Permeability for large molecules was quantified by counting the number of leaky sites (leaks—visible extravascular spots with diameter>100 μm) in the prepared area (1cm^2^) using an UV-light microscope (40× magnification). The number of leaks was counted at baseline and during reperfusion at times 0, 5. 10, 15 and 30 min (T_0_, immediately after; T_5_; 5 min after and T_10_ and so on; 10 min after tourniquet release is the time when maximum number of leaks is seen. Preparations presenting more than 10 spontaneous leaky sites or petechiae at baseline were excluded due to previous damage to the tissue. In order to evaluate histamine-mediated vascular permeability, histamine solution was topically applied on the preparation, after a 30-minute washout-period (5 minutes topical application of 200μl/min; 5 μM; Histamine dihydrochloride, Sigma Chemicals, St. Louis, MO, USA). The number of leaks was counted at baseline and at 2, 5, 10, 15, 20 and 30 min after the beginning of histamine application and results are reported using values obtained at 5 min.

### 3.8—Analytic methods

Immediately after the sacrifice, approximately 2.5 ml of blood was collected, centrifuged [3000 r min−1 (1157 ×g) for 10 min at 4°C], and stored at −80°C. Total insulin concentration was assayed by radioimmunoassay (BioTrak, Amersham-Pharmacia Biotech, Piscataway, NJ, USA). Glucose levels were measured in the blood drawn through saphenous vein puncture [21], using a capillary tube containing heparin (Heparina Perfecta, 75mm length tubing; São Paulo, SP,—Brazil, One touch ultra—Johnson e Johnson, Medical Brazil).

### 3.9—Statistical analysis

Results are presented as mean±SD unless otherwise noted. Microvessel diameters are presented as relative changes from baseline. Data between groups were analyzed using One Way ANOVA and post-hoc Newman-Keuls tests. Group differences in arteriolar diameter responses to Ach and SNP were determined by repeated ANOVA measures. Statistical analysis was performed using Prism 5.0 (Graphpad Inc., San Diego, CA, USA). Results were considered statistically different when p<0.05.

## Results

### Weight evaluations

In order to investigate whether the caloric overload provided by fructose solution elicited a higher increase in body weight, animals were weighed every two weeks. Interestingly, no significant difference between treated groups could be detected, with either fructose or vitamin E. The average fructose intake was 4 mg/kg/day and the caloric intake did not differ between animals, but those drinking 10% fructose solution showed a trend towards lower consumption (Table 2).

### Biochemical markers and hemodynamic parameters

Analyses of collected blood on euthanize day showed higher glucose and insulin levels in all fructose-drinking groups, regardless of vitamin E treatment or concentrations. No significant differences on triglycerides, cholesterol or hemodynamics parameters could be detected (Table 2).

### Microvascular reactivity

#### Endothelium-dependent responses

**Fig 2:**
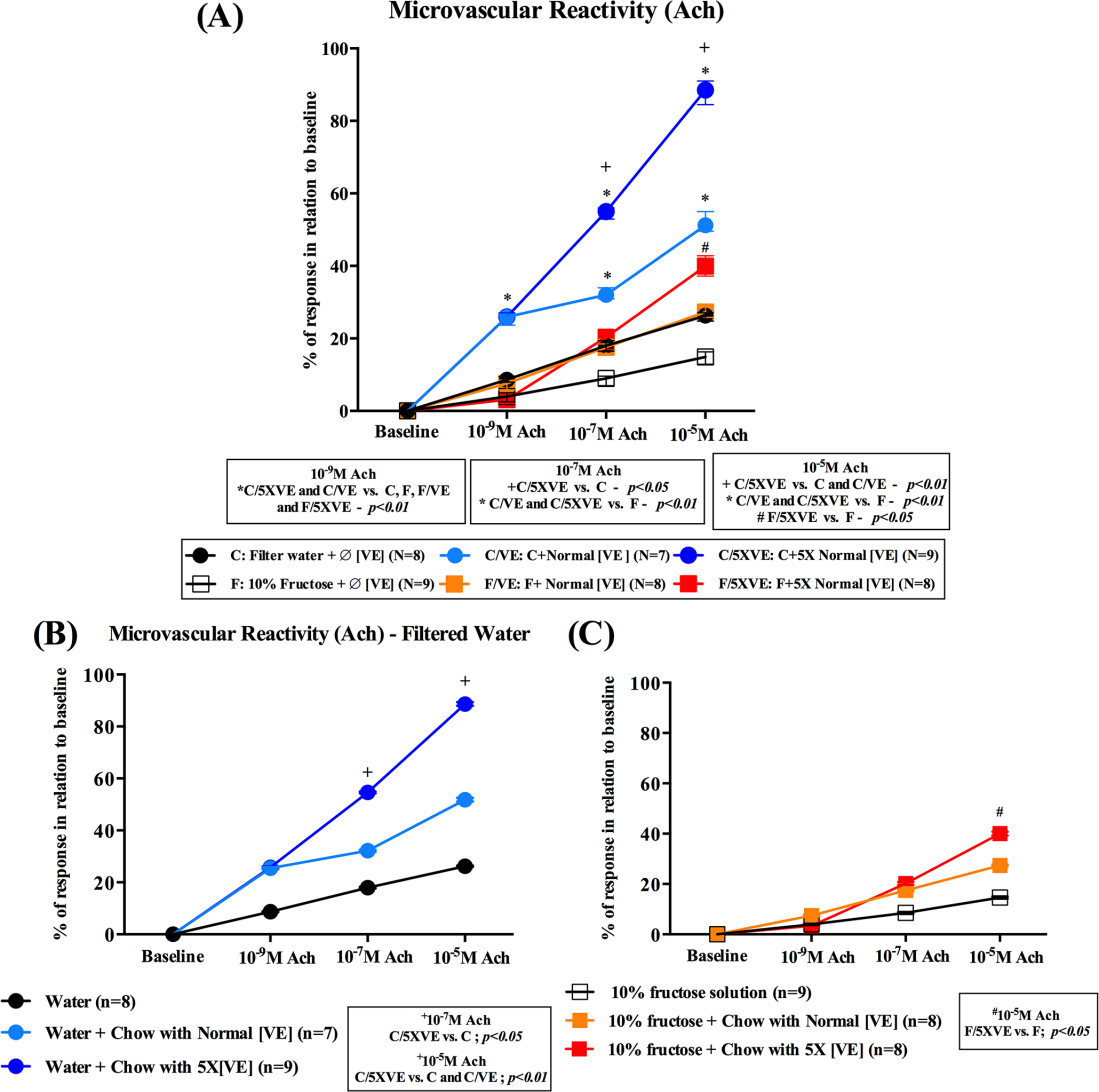
Mean arteriolar diameters after topical application of Ach–Endothelial- dependent evaluation. Data are shown as changes of average diameter, expressed as mean ± SD and plotted in superimposed symbols. **(A)** Overall mean arteriolar diameters after topical application of three concentrations of acetylcholine (10^−9^, 10^−7^ and 10^−5^ M) in the 2 treated groups. **(B)** Endothelial-dependent responses of groups treated with filtered water, concomitantly associated with chows without vitamin E (C), with normal concentration of vitamin E (75U/kg–C/VE) and supplemented concentrations of vitamin E (375U/kg–C/5XVE). **(C)** Endothelial-dependent responses of groups that had the filtered water substituted by 10% fructose solution, concomitantly associated with chows without vitamin E (F), with normal concentration of vitamin E (75U/kg–F/VE) and supplemented concentrations of vitamin E (375U/kg–F/5XVE). ^**+**^Significantly different in relation to control; ^+^ *p*<0.05 [10^-7^M] and both control and control with normal concentration of vitamin E; ^+^ *p*<0.01 [10^-5^M]. ^#^Significantly different from fructose-drinking without vitamin E in chow (^#^ *p*<0.05).

Fructose ingestion resulted in significant alterations of endothelial cell function, observed by decreased vasodilatation to acetylcholine (ACh–Fig 2), in all concentrations topically applied. Although the treatment with vitamin E did not change the relationship between control and fructose-drinking responses, it was possible to observe an increment in the responses dependent on vitamin E concentration in the fructose-drinking group [Ach 10^-5^M - (F/5XVE vs. F– 63.9% difference in the response) and (F/5XVE vs. F/VE– 31.7% difference in the response); Fig 2C]. Moreover the amelioration of the response was not limited to fructose-drinking animals, but the control groups also showed an improvement in endothelial function dependent on vitamin E concentration [Ach 10^-5^M (C/5XVE vs. C– 70.7% difference in the response) and (C/5XVE vs. C/VE– 41.8% difference in the response).

#### Endothelium-independent responses

**Fig 3:**
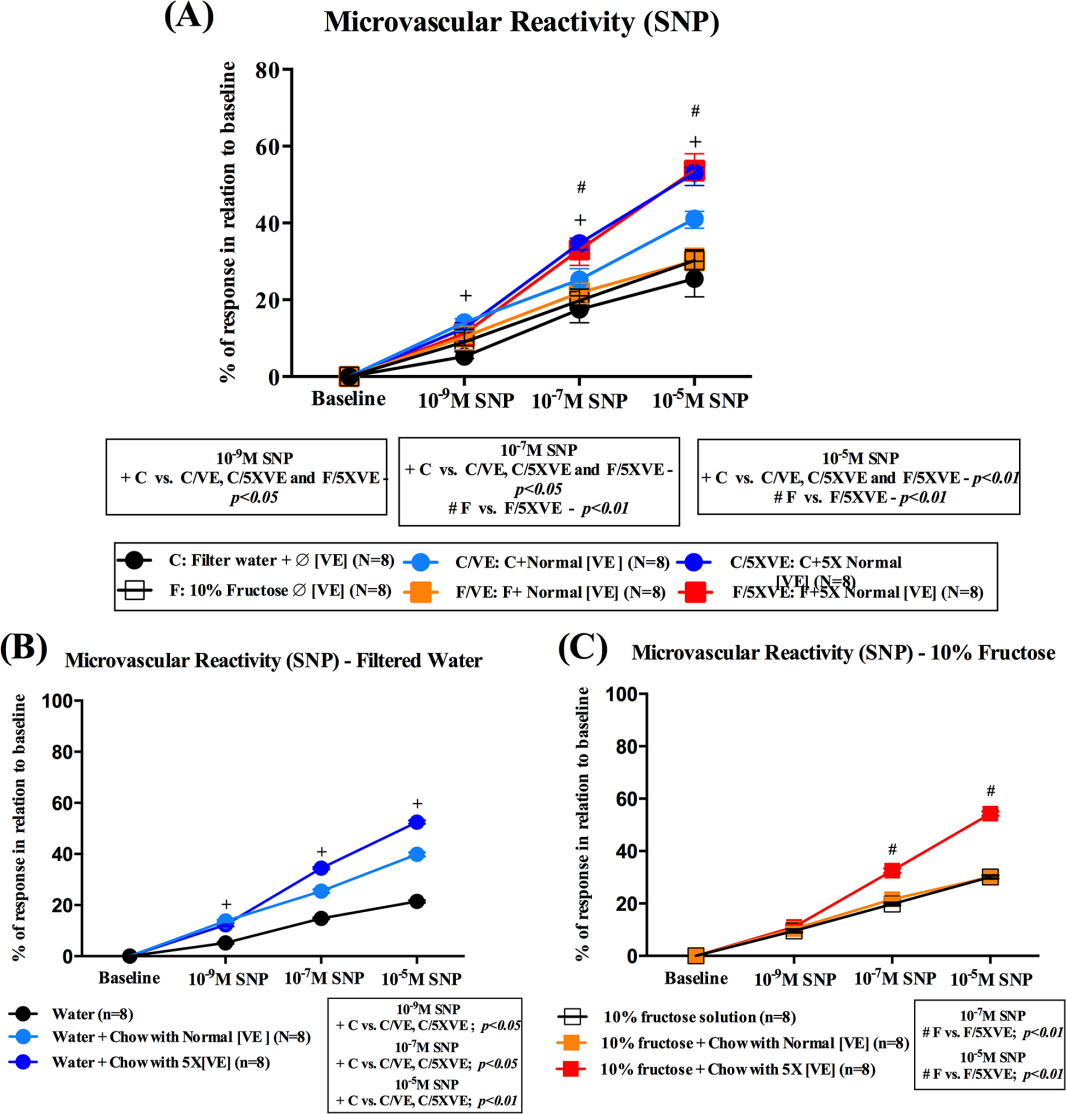
Mean arteriolar diameters after topical application of SNP–Endothelial- independent evaluation. Data are shown as changes of average diameter, expressed as mean ± SD and plotted in superimposed symbols. **(A)** Overall mean arteriolar diameters after topical application of three concentrations of sodium nitroprusside (10^−9^, 10^−7^ and 10^−5^ M) in the 2 treated groups. **(B)** Endothelial-independent responses of groups treated with filtered water, concomitantly associated with chows without vitamin E (C), with normal concentration of vitamin E (75U/kg–C/VE) and supplemented concentrations of vitamin E (375U/kg–C/5XVE). **(C)** Responses of groups that had the filtered water substituted by 10% fructose solution, concomitantly associated with chows without vitamin E (F), with normal concentration of vitamin E (75U/kg–F/VE) and supplemented concentrations of vitamin E (375U/kg–F/5XVE). ^**+**^ *p*<0.05 [10^-9^M and 10^-7^M] and *p*<0.01 [10^-5^M]. Significantly different from Control without vitamin E and ^**#**^p<0.01 significantly different from fructose-drinking solution without vitamin E.

Impairments were not limited to endothelial function, but to microvascular function as a whole, since endothelium-independent responses were also decreased after sodium nitroprusside (SNP) application (Fig 3A). The impairment was however limited to animals treated with chows without vitamin E or with normal concentration of it (75U/kg–F/VE) since higher supplementation with vitamin E restored the normal responses on F/5XVE group (treated with 375U/kg of vitamin E) [SNP 10^-5^M (C/5XVE vs. C– 15.6% decrement of the response) and (C/5XVE vs. C/VE– 26.4% decrement of the response)]. Vitamin E was also responsible for improvements on overall microcirculatory responses, since both fructose-drinking [SNP 10^-5^M (F/5XVE vs. F–increment of 42.5%) and (F/5XVE vs. F/VE–increment of 44.5%)] and control [SNP 10^-5^M (C/5XVE vs. C–increment of 51.5%) and (C/5XVE vs. C/VE–increment of 22%)] groups had increased responses when ingested chows with supplemented contents of vitamin E (Fig 3B and 3C).

### Microvascular permeability to macromolecules

**Fig 4:**
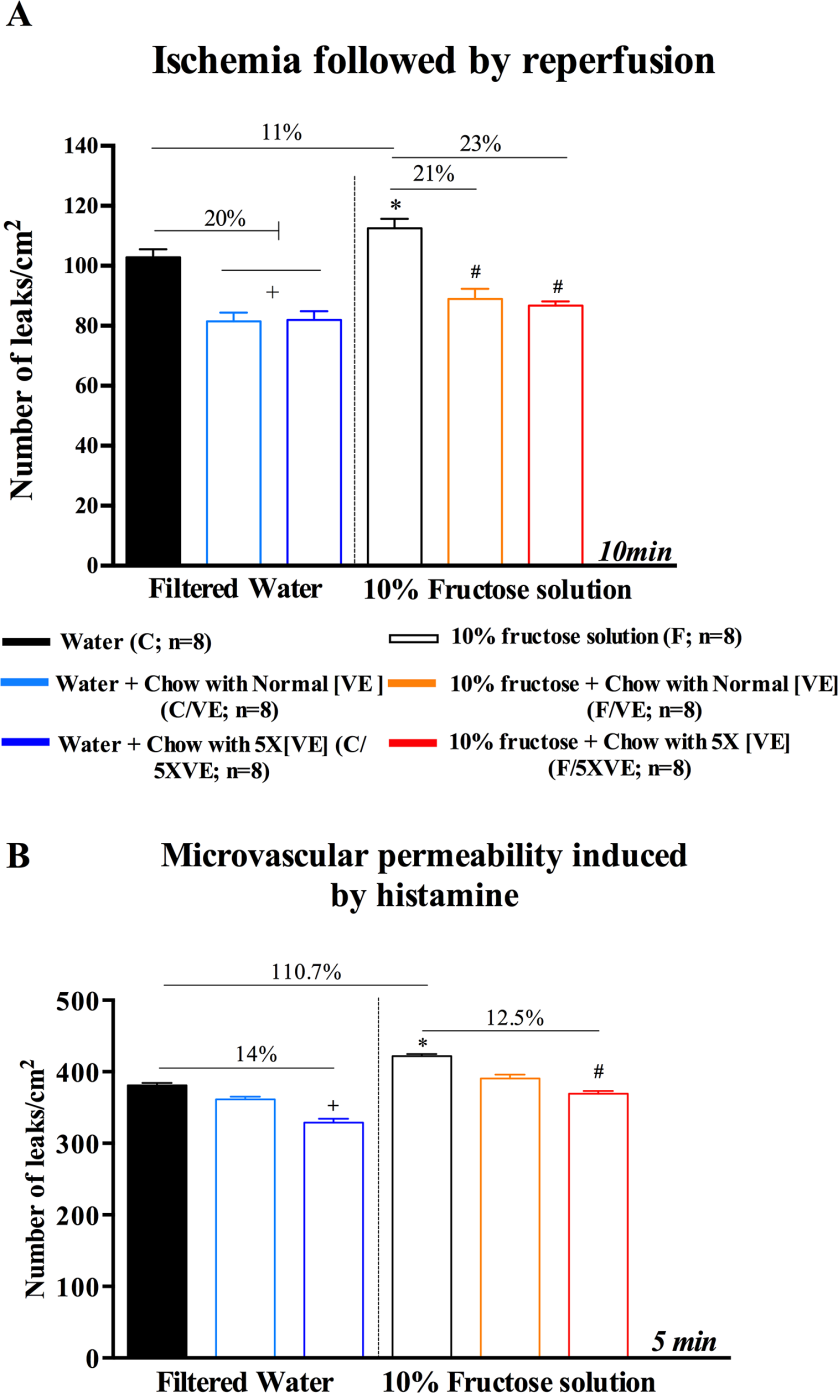
Microvascular permeability measurements after I/R procedure and topical application of histamine. Data are shown as number of leaks, expressed as mean ± SD and plotted in column bars. **(A)** Number of extravasations after 30 min ischemia followed by reperfusion, 10 minutes after the onset of reperfusion. Significant differences were found between *C vs. F (*p*<0.01), + C vs. C/VE and C/5XVE (*p*<0.001), and F vs. F/VE and F/5XVE (*p*<0.01). **(B)** Histamine-mediated microvascular permeability. Animals treated with 10% fructose solution without vitamin E in the chow presented higher number of leaky sites, when compared to controls (C—filtered water without vitamin E in the chow) (^*^ *p*<0.01- F vs. C). Supplementation with vitamin E restored microcirculatory function, as improvements may be seen on both fructose (^**#**^ *p*<0.01 –F vs. F/5XVE) and control responses (^**+**^ *p*<0.01 –C vs. C/5XVE).

Microvascular permeability induced by I/R and histamine also presented significant differences between the 2 groups investigated. Improvements on both ischemia/reperfusion and histamine-induced macromolecular permeability are illustrated on Fig 4A and 4B. The number of leaky sites after I/R procedure in fructose-drinking/without vitamin E animals showed an increase of only 11%, compared to its paired controls (*p<0.05 –C vs. F), while more substantial improvement occurred when supplementation with vitamin E was added. Controls and 10% fructose-drinking animals with normal and supplemented chows had significant decrease in the number leaky sites, in the order of 20% in control (*p<0.01 –C vs. C/VE and C/5XVE), and (F/VE) and (F/5XVE) in fructose-drinking groups (^#^ *p*<0.01 –F vs. F/VE and F/5XVE).

## Discussion

In our investigation it was possible to demonstrate that substitution of the drinking water by 10% fructose solution, in hamsters, elicited hyperglycemia, hyperinsulinemia, endothelial dysfunction and increased microvascular permeability for macromolecules. This scenario could be modified by feeding the animal with chow containing normal or supplemented concentrations of vitamin E, suggesting that it could be a therapeutic approach to reverse microvascular alterations.

To evaluate dietary modification effects on the microvasculature, the chosen microcirculatory assessment technique was the hamster cheek pouch preparation that displays robust, stable blood flow and can respond to topic application of vasoactive drugs. Its transparence based on translucent conjunctive tissue makes it very practical to check *in vivo*, in real-time, microvascular responses and due to its long stability (5-6h) guarantee that several evaluations may be performed in the same animal (i.e. microvascular reactivity and macromolecular permeability) [17,19]. To have the possibility of applying vasoactive drugs topically is an important issue when one wants to evaluate microvascular reactivity because it is possible to avoid their systemic effects (like significant changes in mean arterial pressure) that could mask their effects on the microcirculation. Although the microcirculatory bed observed in the cheek pouch during this investigation was mostly the cutaneous part of it, it is conceivable to consider that it represents systemic alterations. Experiments in humans using laser Doppler and intravital videomicroscopy to examine the skin of the dorsum of the middle finger have shown a good correlation between cutaneous microcirculation and systemic alterations [22]. Microvascular alterations observed on cutaneous tissue attributed to microvascular dysfunction are not only local, but also a systemic process, and studies concerning the cutaneous vascular bed may reproduce alterations on muscle tissues [23,24]. Microvascular dysfunction is associated to co-morbidities such as diabetes and hypertension and could be considered as consequence of reduction in vasodilator bioavailability, like nitric oxide, and activated endothelial cells.

Effects of chronic administration of fructose solution on body weight are contradictory in the literature, varying from augmentation [9,25] to no significant changes [26]. Félétou and co-workers, using the cheek pouch preparation and substitution of the drinking water by 10% fructose solution for 18 weeks, did not find a significant increase on body weight compared to control hamsters either [26]. Body weight measurements varied between treated animals since, as exposed on Table 2, fructose feeding reduced chow intake as satiety is reached sooner than in animals ingesting filtered water. This limitation might stand as a deadlock for vitamin E treatment in this particular experimental design. Additionally, peculiarities between palatability of animals themselves, concerning fructose ingestion, were found responsible for some variation within body weight of fructose-drinking animals. Addition of vitamin E to the diet, as expected, did not influence body mass of treated hamsters, since α-tocopherol stands for a non-caloric micronutrient. Fructose was, therefore, considered the only added energy-giving substance in the diet capable to exert effects on animals’ body mass.

Plasma insulin levels increased in all fructose groups and it plays an important role on increasing capillary recruitment via increase in NO bioavailability and muscle perfusion [27]. This insulin-induced vasoreactivity is crucial not only to deliver nutrients that could match tissue’s needs but also for its action in terms of whole-body insulin sensitivity [28–30], however a functional endothelium is necessary to elicit vasodilator effects on nutrients arterioles. Insulin’s vasoactive properties initiates both vasodilator and vasoconstrictor responses, the former through activation of insulin receptor followed by Akt and nitric oxide synthase (eNOS) with consequent nitric oxide (NO) production [31,32] and the second one through extra-cellular signal-related kinase 1/2 (ERK1/2) phosphorylation with subsequent production of endothelin-1 [33]. The pathogenesis of type 2 diabetes appears to involve at least 2 defects in this regulatory system. When insulin vasoactive actions are jeopardized, the normal balance that favors NO production is shifted towards ET-1 overproduction, leading to blunted insulin-induced vasodilatation in postprandial states. The earliest detectable lesion of this vicious circle is insulin resistance in peripheral tissues [34] progressing to hyperinsulinemia and hyperglycemia. In our investigation, animals drinking 10% fructose solution showed significant increase on glucose and insulin levels, similar to findings reported by Hwang and co-workers [35]. Blood vessels chronically exposed to hyperglycemia have as outcome decreased vasodilator capacity since endothelium integrity is harmed. Additionally oxidative stress induced by hyperglycemia is one of the key factors in the pathogenesis of diabetes complications [36]. Glucose overload damages to cells occur through oxidative stress responses and vitamin E treatment seems to either suppress or attenuate those effects. Thus, disturbances on glucose and lipid metabolism could be important factors to impair NO bioavailability and its vasodilator effects [27]. Several mechanisms might be involved on microvascular impairment in type 2 diabetes, like for instance, chronic reduction of NO bioavailability secondary to increased oxidative stress [37] and/or reduction in both convective O_2_ delivery and diffusive O_2_ transport [38], increased activity and expression of protein kinase C [39] and/or reduction in tetrahydrobiopterin (BH4) [40]. Addition of Vitamin E did not improve glucose or insulin levels, independently of its concentration, nevertheless our in vivo findings point to an effect on the microcirculation itself since both endothelial function and macromolecular permeability barrier were ameliorated.

Fructose elicits microvascular dysfunction since all fructose-drinking hamsters presented endothelial responses significantly different than the ones kept drinking filtered water [25,41]. It was possible to observe endothelial and microvascular dysfunction in view of decreased responses to acetylcholine and sodium nitroprusside in all F groups compared to C ones. With the ingestion of vitamin E, F/5XVE hamsters stand out as the group with greater improvements on microcirculatory dysfunction, reflected by augmented responses to Ach, an endothelium-dependent (at 10^−5^ M) and SNP, and endothelium-independent (at 10^-7^M and 10^-5^M) vasodilator, compared to FRU and FRU/VE ones (Figs 3 and 4), suggesting that supplementation with an antioxidant could be considered as treatment to microvascular damages provoked by sugar overload. These data corroborate with the ones found in the literature, where acute and chronic supplementation with antioxidants, such as vitamin C and E improved endothelial function in hypertensive patients [42], chronic heart failure [43] and diminished endothelial dysfunction in elderly [44]. Although further molecular investigations are necessary, the mechanism associated with these improvements could probably be related to rise on NO availability and activity of the enzyme nitric oxide synthase (eNOS). Hyperglycemia-induced reduction in NO release in streptozotocin-injected rats could be prevented by treatment with vitamin E [45], pointing to endothelium NO production as responsible for observed improvements.

Another important evaluation in the present investigation was the macromolecular permeability increase induced by ischemia/reperfusion or histamine due to the possibility presented by this preparation to analyze microvascular morphophysiological alterations caused by these agents [46]. Animals chronically drinking 10% fructose solution (F, F/VE and F/5XVE) showed higher number of leaky sites when compared to C ones, after ischemia/reperfusion and topical application of histamine, demonstrating the aggressive oxidative effect of sugar overload on the microcirculation. However, the use of supplemented chow with vitamin E (F/5XVE) reduced the number of leaky sites, suggesting that vitamin E may act as antioxidant defense against inflammatory responses [47]. Vitamin E has long been recognized as one of the major natural antioxidant [48,49] and Wistar rats chronically drinking 10% fructose solution and treated with vitamin E have shown significant improvements on insulin sensitivity, some components of the antioxidant defense system [50], blood pressure, cardiac collagen deposition, and ventricular stiffness [51]. However our observations pointed to the necessity of supplementation with vitamin E to achieve better microcirculatory responses, inasmuch as normal concentration of vitamin E was not sufficient to reverse damages once microvascular dysfunction is installed. The same statement may not be repeated concerning control animals; in these ones normal to supplemented concentrations of vitamin E were sufficient to improve microvascular responses, reinforcing our findings that previous damages require higher amounts of antioxidants. Evaluations of micro- and macrocirculation of type 1 and 2 diabetic patients showed no significant improvement after long-term treatment with vitamin E, indicating that supplemented levels are necessary under disturbed microcirculatory environments [52].

We realize that our study has limitations, such as lack of antioxidant defenses machinery analysis since the use of hamsters as an animal model, though enable microcirculatory assessments, does not allow serum and plasma analysis due to absence of viable kits for this species. Considering the impossibility of performing such analysis, one could only infer that these defenses were augmented in vitamin E treated groups, via our microcirculatory evaluations. Additionally, due to logistic issues, vitamin E was administrated through the chow, which hinders the certainty that all hamsters ingested the same daily amount of this substance, consequently influencing the homogeneity of the groups.

In conclusion, we have showed that supplementation with α-tocopherol substantially improved microvascular dysfunction observed on fructose-drinking hamsters.
